# Vegetarians have an indirect positive effect on sleep quality through depression condition

**DOI:** 10.1038/s41598-023-33912-7

**Published:** 2023-05-03

**Authors:** Xiaodi Wang, Fangfang Song, Bian Wang, Lei Qu, Zhiping Yu, Xiuhua Shen

**Affiliations:** 1 Department of Nutrition, School of Public Health, Shanghai Jiao Tong University School of Medicine, Shanghai, People’s Republic of China; 2 Department of Clinical Nutrition, College of Health Science and Technology, Shanghai Jiao Tong University School of Medicine, Shanghai, People’s Republic of China; 3 Department of Nutrition and Dietetics, Brooks College of Health, University of North Florida, Jacksonville, FL USA; 4 Shanghai Key Laboratory of Pediatric Gastroenterology and Nutrition, Xinhua Hospital Affiliated to Shanghai Jiao Tong University School of Medicine, Shanghai, People’s Republic of China; 5 Department of Clinical Nutrition, Xinhua Hospital Affiliated to Shanghai Jiao Tong University School of Medicine, Shanghai, 200092 People’s Republic of China

**Keywords:** Risk factors, Health care, Nutrition, Public health

## Abstract

This study aimed to assess the association between a vegetarian diet and sleep quality among Chinese healthy adults and explore potential contributing factors. A cross-sectional study was conducted with 280 vegetarians and 280 age- and sex-matched omnivores from Shanghai, China. The Pittsburgh Sleep Quality Index (PSQI) and the Central Depression Scale (CES-D) were used to assess sleep and depression condition, respectively. A validated semi-quantitative food frequency questionnaires (SQFFQ) was employed to assess dietary intakes, and body composition was measured with InBody720. Multi-linear regression and logistic regression analysis were performed for the data analysis. The sleep quality was significantly better in the vegetarians than in the omnivores (PSQI score: 2.80 ± 2.02 vs. 3.27 ± 1.90, *p* = 0.005). The proportion of vegetarians who reported self-satisfied sleep was also higher than that of the omnivores (84.6% vs. 76.1%, *p* = 0.011). However, after adjusted for the depression condition (CES-D scores), the difference in sleep quality between vegetarians and omnivores became insignificant (*p* = 0.053). Compared to omnivores, vegetarians had lower depression scores (CES-D: 9.37 ± 6.24 vs. 10.94 ± 7.00, *p* = 0.006). After controlling for confounding factors, there was positive association between depression condition and sleep quality (β = 0.106, 95%CI: 0.083 to 0.129, *p* < 0.001). Similarly, participants with better CES-D score had a lower risk of sleep disorders after controlling for the same confounding factors (OR = 1.109, 95%CI: 1.072 to 1.147, *p* < 0.001). Different contributing factors were reported in the vegetarian group and omnivore group. In conclusion, a vegetarian diet might improve sleep quality by moderating mental health, particularly depression condition.

## Introduction

Sleep quality refers to how well an individual is sleeping, i.e. whether the sleep is restful and restorative. It is important for physiological and psychological functions, such as restoring brain energy stores and optimal performance, and increasing attention and creativity^1^. Poor sleep quality problems including sleep fragmentation, sleep continuity disturbance, circadian dysrhythmia can be defined as sleep disturbances^2^. Sleep disturbances, on the other hand, can cause many adverse health-related consequences, e.g. the augmentative burden of diseases and risk of mortality from cardiovascular diseases and other diseases^3,4^. Due to the outbreak of Coronavirus Disease 2019 (COVID-19), a web-based cross-sectional survey showed that the overall prevalence of sleep quality of the public has reached 18.2% in China^5^. Thus, it is critical to identify lifestyle strategies to improve sleep outcomes in the general population. In addition to voluntary sleep restriction, there are a variety of factors that impact the quality of sleep, including depression condition, sleeping environment, age, sex, sedentariness, caffeine intake and diet^6–9^. Given the complex interactions of sleep, depression, and other behaviors, a bidirectional causal relationship between sleep and depression is widely accepted throughout the body of literature in this field^10^. In our study, the roles of these factors on sleep were taken into consideration as much as possible to study the association between vegetarian diet and sleep. Vegetarianism is a diet derived from plants, and excluding any animal meat, with or without the use of dairy products, eggs and/or honey^11^.

Dietary patterns generally describe long-term diet structure and habits, which have been shown to influence sleep quality and duration^12,13^. For example, previous studies in China and other countries have showed that high-carbohydrate intakes were associated with reduced sleep onset latency and slow wave sleep, as well as increased rapid eye movement, whereas high-fat intakes reduced sleep efficiency and were associated with higher arousals^14^. In a Indiana randomized controlled trial, sleep quality was found to be improved in obese or overweight participants following four weeks of an energy-restricted diet with 20% protein intake, compared to 10% and 30% protein intakes^15^. Moreover, some other studies have reported the sleep-promoting effects of specific foods e.g. milk, fatty fish, tart cherry juice, and kiwifruit^14,16^. However, there have been few studies to assess the association between vegetarian diets and sleep health. Vegetarian diets are usually low in total and saturated fat^17^, which may improve sleep quality. One study by Daneshzad, E. et al. demonstrated that diabetic participants who adhered to plant-based diets rather than animal-based diets (judged by plant-based diet indices, a higher intake of indices reflects lower animal food intake) were more likely to ensure good sleep assessed by the Pittsburgh sleep quality index (PSQI)^18^. In this study, we aimed to examine the effects of vegetarian diets and associated factors on sleep quality of healthy adults (participants aged 18–60 without nutritional malabsorption or systemic diseases).

## Methods

### Study participants

A total of 560 adults aged 18–61 years were recruited in Shanghai, China between the period of March 2016 and May 2016, including 280 vegetarians and 280 omnivores. The inclusion criteria of vegetarians were as follows: (1) age at least 18 years; (2) resided in Shanghai for more than half a year; (3) adopted a vegetarian diet for at least a year; (4) understand the contents of questionnaires. Participants with severe nutritional malabsorption or systemic diseases or pregnant within the preceding 12 months were excluded. Omnivores were recruited among the vegetarians’ relatives and friends who were matched respectively for the same gender and age (± 1 year) with vegetarian participants. The written informed consent procedures were accordingly performed with the latest revised Declaration of Helsinki (2013). This study was approved by the Institutional Review Board of the Shanghai Jiao Tong University School of Medicine, and we confirmed that all research was performed in accordance with relevant guidelines and regulations.

### Assessment of sleep quality

A modified Chinese version of the PSQI scale was used to determine participant’s subjective sleep quality^19^. The PSQI scale is a well validated, widely used tool to measure sleep quality. It includes a total of 14 self-rated items combined to form seven component scores (i.e., subjective sleep quality, sleep latency, sleep duration, habitual sleep efficiency, sleep disturbances, use of sleeping medications, and daytime dysfunction), each of which has a range of 0–3 points^20^. The sum of these seven PSQI component scores is the total PSQI score, with higher scores indicating poorer sleep quality. Our minor modification included adding the part of daytime dysfunction (effects of sleep on daytime mood and daytime sleepiness) and eliminated questions of sleep disturbances (whether you feel cold or hot when you sleep). Meanwhile, one more question about “Do you feel satisfied with your sleep?” was also included in the questionnaire, which measured the participants’ “sleep self-satisfaction”.

### Assessment of depression condition

The Center for Epidemiologic Studies Depression Scale (CES-D) measures symptoms of depression and a scoring ≥ 16 (range 0–60) was considered as “at risk for clinical depression^21^”. Participant at risk for clinical depression has a higher probability of being diagnosed as depression by clinicians. The higher total score indicates a greater risk of depression. In addition to the total score, the CES-D includes 4 subscales: depressed affect (range 0–21), positive affect (range 0–12), somatic (range 0–21), and interpersonal (range 0–6). The CES-D has been validated in Chinese population^22^. Favorable reliability was demonstrated in suicide attempters and residents with Cronbach’s alpha values of 0.940 and 0.895^23^.

### Anthropometric and dietary assessments

Participants’ demographics and personal behavior information, including age, gender, income, alcohol consumption, smoking, physical activity, sedentary time, vegetarian categories (vegan or lacto-ovo-vegetarian), and practicing vegetarianism duration, was assessed through a general condition questionnaire. Specifically, smoking habit included frequency of smoking and age of starting smoking, alcohol consumption investigated the frequency and amount of drinking, as well as the types of alcohol consumed. Physical Activity investigated the frequency and duration of physical activity in spare time, as well as the most commonly used forms of exercise. Vegetarians were classified as people who exclude any animal meat, with or without the use of dairy products, eggs and/or honey for at least 1 year; otherwise, they were classified as omnivores^11^. Vegans exclude any use of any animal products for any purpose, and lacto-ovo-vegetarians were defined as those who eat plant food plus eggs and milk products^11^.

Participants’ body composition including body fat percentage (%), fat mass (kilograms), skeletal muscle mass (kilograms), and total body weight (kilograms) was assessed using InBody720 (brand: *Biospace, Korea*). Participants' height, body weight, waist circumference, and hip circumference were obtained from the physical examination by trained dietitians in accordance with the standardized protocol. Body weight was measured in minimal clothing using InBody720. Height, waist circumference, and hip circumference were measured while participants were in standing position using an unstretched tape measure to the nearest to 0.1 cm. Skeletal muscle percentage (%) is the percentage of skeletal muscle mass to total body mass and was calculated as skeletal muscle mass (kilograms) divided by body weight (kilograms). Body mass index (BMI) was calculated as weight (kilograms) divided by height (meters) squared. BMIs of 24 kg/m^2^ and 28 kg/m^2^ were used as the cut-offs for overweight and obesity, respectively, according to the criteria for Chinese adults^24^. Waist-to-hip ratio was calculated as the measured waist circumference divided by the measured hip circumference.

Dietary intake was collected by well-trained dietitians, using a 112-item, validated semi- quantitative food-frequency questionnaire (SQFFQ), which was used in 2002 China Nutrition and Health Survey (CNNHS)^25^. This SQFFQ contains 112 food categories, which were divided into 13 food modules, namely: 1 grains, potatoes, miscellaneous beans; 2 beans; 3 vegetables; 4 bacteria and algae; 5 fruits; 6 milks; 7 eggs; 8 nuts; 9 beverages; 10 meats; 11 oils; 12 snacks; and 13 condiments. Participants were requested to recall the frequency of 112 food items over the previous 12 months and the estimated portion size, using weight units (g) or volume units (ml). For each food item, the consumption frequency was reported using 5 categories in this questionnaire, ranging from “never”, “times /year”, “times/month”, “times/week”, “times/day”. During face-to-face interviewing, oral descriptions, food images, and food models were provided by dietitians to help the participants recall and estimate their dietary intake. Then, the frequency of intake and portion size were used to calculate the amount of each food item consumed on average, and converted into a daily intake, using the Chinese Food Composition Table^26^ as the database. Daily nutrient intakes were calculated by our self-designed “FFQ nutrient calculator” using Visual Basic language in Excel 2002 software. As coffee intake and tea intake reflect the level of caffeine intake, which are important factors in sleep quality, they were selected as part of dietary intake.

### Statistical analysis

All statistical analyses were performed using SPSS version 22.0 software (SPSS Inc., Chicago, IL, USA), and two-sided p values < 0.05 were considered as statistically significant. The continuous variables were shown as mean ± standard deviation (SD), while the categorical variables were expressed as percentage (%). To compare the differences between vegetarians and omnivores, after checking the normality of the variables, the continuous data were analyzed by paired Student’s *t* test or paired non-parametric test respectively, according to whether the normality was met or not. And paired chi-square test was used for categorical variables.

Variables met the assumption for multiple linear and multiple logistic regression. Multivariable adjusted β coefficients [95% confidence intervals (CIs)] and p values for the association of CES-D score and whether following a vegetarian diet were estimated by linear regression, controlling for age, sex, education, marital status, income, work intensity, physical activity time, alcohol consumption, smoking habit, sleep quality (PSQI score), systolic blood pressure (mmHg), BMI. These factors are usually identified as risk factors or protective factors for depression^27,28^. Multivariable adjusted β coefficients [95% confidence intervals (CIs)] and p values for the associations of sleep quality and its influencing factors were estimated by linear regression and logistic regression. The different models in the multi-linear regression and logistic regression were as follows: Model 1, unadjusted regression; Model 2, regression with age, sex, income, alcohol consumption, smoking habit, working intensity, physical activity and sedentary time controlled; Model 3, daily dietary intakes (energy, fat, protein, carbohydrate, and fiber), frequency of drinking coffee or tea controlled building upon model 2. Among them, work intensity refers to the physical intensity of work, while physical activity is the length of physical exercise in spare time each week. These factors are generally considered to be important factors affecting sleep quality, which was identified by consulting the literature^29–31^. For example, a meta-analysis of sleep studies across the human lifespan showed that sleep duration and efficiency reduced as age increased^32^. A systematic review of sixteen eligible studies revealed prolonged sedentary behavior tends to be associated with an elevated risk of insomnia and sleep disturbance^9^. Control for different variables. Therefore, these variables were controlled as confounding factors.

## Result

### Characteristics of subjects

Table 1 represents the general characteristics of the total samples and vegetarians and omnivores participants. A total of 280 vegetarians and 280 matched omnivores were included in the study. In both groups, the majority of participants was females (82.5%). Comparing to omnivores, vegetarians had a lower BMI, waist-to-hip ratio values, alcohol consumption, daily intakes of energy, protein, and fat, (all *p* < 0.05), but higher income, physical activity level and daily intakes of fiber (all *p* < 0.05). In addition, vegetarians had significantly lower PSQI total score than omnivores (2.80 ± 2.02 vs. 3.27 ± 1.90, *p* = 0.005).

**Table 1:** Characteristics of vegetarians and omnivores.

	Vegetarian (n = 280)	Omnivore (n = 280)	*p*
Age (y)	34.93 ± 8.74	34.44 ± 8.85	0.457
Female, %	82.5	82.5	1
Male, %	17.5	17.5	1
Vegetarian duration (y)	5.61 ± 5.04	–	
Nonsmoker, %	89.6	92.5	0.206
No alcohol, %	94.6	82.1	< 0.001*
Ethnicity, %			
Han	95.3	93.9	0.173
Else	4.7	6.1	
Working intensity, %			
Light	87.9	88.9	0.997
Middle	8.2	6.5	
Heavy	3.9	4.7	
Income (Yuan/month/person), %			
< 3000	19.4	25.0	0.014*
3000–8000	43.0	45.9	
8000–15,000	24.4	20.8	
> 15,000	13.2	8.2	
Physical activity (min/w)	114.69 ± 148.08	82.48 ± 117.16	0.010*
Sedentary time	8.11 ± 3.78	8.74 ± 4.88	0.214
Body composition			
BMI (kg/m^2^)	21.03 ± 2.59	22.36 ± 3.30	< 0.001*
Waist-to-hip ratio	0.82 ± 0.05	0 .84 ± 0 .05	< 0.001*
Fat mass (%)	24.95 ± 8.44	27.95 ± 7.10	< 0.001*
Skeletal Muscles	22.29 ± 4.07	23.19 ± 4.82	< 0.001*
Dietary intake			
Energy (Kcal/d)	1525.98 ± 525.31	1744.74 ± 580.29	< 0.001*
Protein (g/d)	47.18 ± 21.00	69.76 ± 32.85	< 0.001*
Fat (g/d)	42.98 ± 22.33	65.20 ± 32.65	< 0.001*
Carbohydrate (g/d)	230.83 ± 88.59	213.82 ± 77.42	< 0.001*
Fiber (g/d)	15.68 ± 9.59	11.90 ± 6.89	0.019*
Coffee intake (ml/d)	38.85 ± 109.04	60.63 ± 133.65	0.003*
Tea intake (g/d)	2.54 ± 7.08	1.96 ± 3.02	0.758
Sleep and mental health			
PSQI score	2.80 ± 2.02	3.27 ± 1.90	0.004*^#^
Sleep self-satisfaction	84.6%	76.1%	0.007*^#^
Sleep duration	452.81 ± 83.18	465.02 ± 52.33	< 0.001*
CES-D score	9.37 ± 6.24	10.94 ± 7.00	0.006*

*BMI* body mass index.

*Statistical significance when comparing vegetarian and omnivore groups (paired *t* test or paired non-parametric analysis).

^#^No statistical significance when assessed with covariance controlling for CES-D score between vegetarian and omnivore groups.

### The relationship between vegetarian diets, sleep quality and depression

After controlling for the factors generally considered to be important factors affecting sleep quality, including gender, age, alcohol consumption, smoking habit, working intensity, physical activity and sedentary time, daily dietary intakes (energy, fat, protein, carbohydrate, and fiber), and frequency of drinking coffee or tea, the proportion of vegetarians’ sleep self-satisfaction was significantly higher than that in the omnivores (84.6% vs. 76.1%, *p* = 0.011). Although vegetarians sleep an average of 12 min less than omnivores, the sleep quality of vegetarians was better than omnivores, suggesting that vegetarians may have higher sleep efficiency. The CES-D score of vegetarians was also better than that of omnivores (9.37 ± 6.24 vs. 10.94 ± 7.00, *p* = 0.006).

However, after controlling for CES-D scores, PSQI scores did not significantly differ between vegetarian and omnivore groups. (Table 1). Likewise, after controlling for CES-D score, the logistic regression analysis showed that there was no significant association between vegetarian diets and “sleep self-satisfaction”.

**Figure 1:**
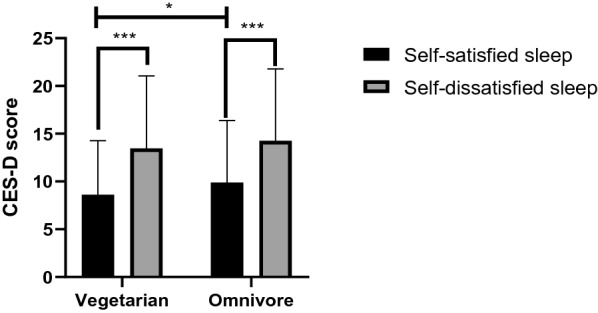
CES-D score of vegetarians and omnivores in different sleep quality groups. In vegetarian and omnivore groups, participants were divided into either self-satisfied sleep subgroup or self-dissatisfied sleep subgroup. In vegetarian group, CES-D scores were significantly lower in self-satisfied sleep subgroup (n = 237) than in the self-dissatisfied sleep subgroup (n = 43, CES-D: 8.62 ± 5.67 vs. 13.47 ± 7.58, *p* < 0.001). And in omnivore group, CES-D scores were significantly lower in self-satisfied sleep subgroup (n = 213) than in the self-dissatisfied sleep subgroup (n = 67, CES-D: 9.89 ± 6.49 vs. 14.27 ± 7.51, *p* < 0.001). Among participants who were self-satisfied with sleep, vegetarians got lower CES-D scores than omnivores (*p* = 0.027).

In each diet group, participants were divided into either self-satisfied sleep subgroup or self-dissatisfied sleep subgroup. (Fig. 1). In vegetarian group, CES-D scores were significantly lower in self-satisfied sleep subgroup (n = 237) than in the self-dissatisfied sleep subgroup (n = 43, CES-D: 8.62 ± 5.67 vs. 13.47 ± 7.58, *p* < 0.001). And in omnivore group, CES-D scores were significantly lower in self-satisfied sleep subgroup (n = 213) than in the self-dissatisfied sleep subgroup (n = 67, CES-D: 9.89 ± 6.49 vs. 14.27 ± 7.51, *p* < 0.001). Among participants who were self-satisfied with sleep, vegetarians got lower CES-D scores than omnivores (*p* = 0.027).

The multiple regression analysis for associations between whether following a vegetarian diet and depression condition (CES-D score) is displayed in Table 2. Being vegetarian was negatively associated with CES-D score; which means lower risk of depression (β =  − 1.568, 95%CI: − 2.668 to − 0.468, *p* = 0.005). Moreover, there was still a negative linear relationship between vegetarian and CES-D score (β =  − 1.184, 95%CI: − 2.297 to − 0.071, *p* = 0.037), after adjusting for age, sex, ethnicity, education, marital status, income, work intensity, physical activity time, alcohol consumption, smoking habit, systolic blood pressure (mmHg), BMI, PSQI score.

**Table 2:** Associations between whether following a vegetarian diet and CES-D score.

		β (95%CI)	*p*
Whether following a vegetarian diet	Model 1	− 1.568 (− 2.668,− 0.468)	0.005
	Model 2	− 1.184 (− 2.297, − 0.071)	0.037

Model 1: Unadjusted regression;

Model 2: Regression with age, sex, education, marital status, income, work intensity, physical activity time, alcohol consumption, smoking habit, sleep quality (PSQI score), systolic blood pressure (mmHg), BMI controlled.

The multiple regression analysis for associations between CES-D score and PSQI score is presented in Table 3. It showed that CES-D scores were positively associated with PSQI scores in all participants (β = 0.106, 95%CI: 0.083 to 0.129, *p* < 0.001), vegetarian participants (β = 0.110, 95%CI: 0.074 to 0.146, *p* < 0.001) and omnivore participants (β = 0.087, 95%CI: 0. 057 to 0.118, *p* < 0.001) after adjusting for age, sex, income, alcohol consumption, smoking habit, working intensity, physical activity and sedentary time, daily dietary intakes (energy, protein, fat, carbohydrate, and fiber) and coffee or tea intake.

**Table 3:** Multiple regression analysis for PSQI score.

	All		Vegetarian (n = 280)		Omnivore (n = 280)	
	β (95%CI)	*p*	β (95%CI)	*p*	β (95%CI)	*p*
CESD score						
Model 1	0.109 (0.087, 0.132)	0.000	0.126 (0.091, 0.161)	0.000	0.092 (0.062, 0.122)	0.000
Model 2	0.107 (0.084, 0.130)	0.000	0.109 (0.074, 0.144)	0.000	0.087 (0.057, 0.117)	0.000
Model 3	0.106 (0.083, 0.129)	0.000	0.110 (0.074, 0.146)	0.000	0.087 (0.057, 0.118)	0.000

Model 1: unadjusted regression;

Model 2: regression with age, sex, income, alcohol consumption, smoking habit, working intensity, physical activity and sedentary time controlled;

Model 3: daily dietary intakes (energy, fat, protein, carbohydrate, and fiber), coffee and tea intake controlled building upon model 2.

The logistic regression analysis for associations between CES-D score and sleep self-satisfaction is displayed in Table 4. The results showed that CES-D score was positively associated with if felt self-satisfied sleep in all participants (OR = 1.109, 95%CI: 1.072 to 1.147, *p* < 0.001), vegetarian participants (OR = 1.138, 95%CI: 1.069 to 1.212, *p* < 0.001), and omnivore participants (OR = 1.107, 95%CI: 1.058 to 1.159, *p* < 0.001) after adjusting for age, sex, income, alcohol consumption, smoking habit, working intensity, physical activity and sedentary time, daily dietary intakes (energy, protein, fat, carbohydrate, and fiber) and coffee or tea intake.

**Table 4:** Logistics regression analysis for sleep self-satisfaction.

	ALL		Vegetarian (n=280)		Omnivare (n=280)	
	OR (95%CI)	*p*	OR (95%CI)	*p*	OR (95%CI)	*p*
CESD score						
Model 1	1.106 (1.071, 1.142)	0.000	1.125 (1.067, 1.185)	0.000	1.090 (1.047, 1.135)	0.000
Model 2	1.107 (1.071, 1.144)	0.000	1.119 (1.056, 1.186)	0.000	1.091 (1.046, 1.139)	0.000
Model 3	1.109(1.072, 1.147)	0.000	1.138(1.069, 1.212)	0.000	1.107(1.058, 1.159)	0.000

Model 1: Unadjusted regression;

Model 2: Regression with age, sex, income, alcohol consumption, smoking habit, working intensity, physical activity and sedentary time controlled;

Model 3: Daily dietary intakes (energy, fat, protein, carbohydrate, and fiber), coffee and tea intake controlled building upon model 2.

### Other factors affecting sleep quality

Multiple linear regression analysis (Table 5) presented the factors affecting PSQI score in the vegetarian and omnivore groups after controlling with age, sex, income, alcohol consumption, smoking habit, physical activity and sedentary time, daily dietary intakes (energy, fat, protein, carbohydrate, and fiber), coffee or tea intake, CES-D score factors. In the vegetarian group, sedentary time, systolic blood pressure, and visceral fat mass were positively associated with sleep quality, while skeletal muscle rate was negatively associated with sleep quality. In the omnivore group however, PSQI was positively associated with age and work intensity.

**Table 5:** Multiple linear regression analysis of factors affecting PSQI scores in vegetarians and omnivores.

	Vegetarian (n = 280)			Omnivore (n = 280)		
	β (95%CI)	Standardized β	*p*	β (95%CI)	Standardized β	*p*
Age						
Model 1	− 0.033 (− 0.060, − 0.006)	− 0.144	0.016	0.024 (0.001, 0.049)	0.112	0.062
Model 2	− 0.008 (− 0.034, 0.018)	− 0.034	0.553	0.026 (0.001, 0.050)	0.120	0.043
Working intensity						
Model 1	− 0.187 (− 0.702, 0.327)	− 0.043	0.474	0.836 (0.375, 1.298)	0.210	0.000
Model 2	0.086 (− 0.395, 0.566)	0.020	0.726	0.923 (0.457, 10.390)	0.232	0.000
Sedentary time						
Model 1	0.132 (0.071, 0.193)	0.247	0.000	0.009 (− 0.037, 0.055)	0.024	0.692
Model 2	0.119(0.058, 0.181)	0.225	0.000	0.027 (− 0.018, 0.072)	0.069	0.236
Fat mass (%)						
Model 1	0.008 (− 0.021, 0.036)	0.032	0.592	0.046 (0.015, 0.077)	0.171	0.004
Model 2	0.024 (− 0.004, 0.052)	0.101	0.094	0.022 (− 0.011, 0.056)	0.083	0.192
Skeletal muscles (%)						
Model 1	− 0.997 (− 6.782, − 6.782)	− 0.020	0.735	− 9.102 (− 14.966, − 3.237)	− 0.180	0.002
Model 2	− 8.734 (− 15.443, − 2.026)	− 0.180	0.011	− 3.030 (− 10.438, 4.377)	− 0.060	0.421
Visceral fat area						
Model 1	0.000 (− 0.009, 0.009)	0.006	0.918	0.004 (− 0.004, 0.011)	0.057	0.341
Model 2	0.012 (0.002, 0.022)	0.159	0.024	− 0.005 (− 0.015, 0.005)	−0.078	0.328
Systolic blood pressure						
Model 1	0.005 (− 0.014, 0.024)	0.033	0.579	− 0.005 (− 0.021, 0.012)	− 0.034	0.578
Model 2	0.022 (0.003, 0.042)	0.141	0.021	8.726E-5 (− 0.017, 0.017)	0.001	0.992
Energy						
Model 1	0.000 (0.000, 0.001)	0.120	0.044	0.000 (− 0.000, 0.000)	0.029	0.628
Model 2	0.001(− 0.001, 0.001)	0.001	0.187	0.001 (− 0.001, 0.001)	0.001	0.204
Fat						
Model 1	0.007 (0.000, 0.014)	0.125	0.037	0.004 (− 0.005, 0.013)	0.052	0.390
Model 2	− 0.005 (− 0.005, 0.015)	0.005	0.350	− 0.003 (− 0.015, 0.009)	0.006	0.656
Carbohydrate						
Model 1	0.002 (− 0.000, 0.004)	0.095	0.113	0.000 (− 0.002, 0.002)	0.008	0.899
Model 2	0.001 (− 0.001, 0.003)	0.059	0.321	0.001 (− 0.001, 0.003)	0.070	0.238
Fiber						
Model 1	0.010 (− 0.011, 0.030)	0.057	0.345	0.006 (− 0.010, 0.021)	0.043	0.473
Model 2	− 0.006 (− 0.031, 0.019)	− 0.034	0.638	0.003 (− 0.016, 0.023)	0.025	0.735

^#^Regression with age, sex, income, alcohol consumption, smoking habit, working intensity, physical activity and sedentary time, daily dietary intakes (energy, fat, protein, carbohydrate, and fiber), coffee and tea intake, CESD score controlled, excluding the analyzed variables itself.

The associations between daily nutrient intakes and sleep quality were also analyzed. Macronutrient intakes, including fat, carbohydrate and total energy, showed no significant association with sleep quality in either vegetarian or omnivore group. However, these macronutrients were positively associated with PSQI scores in all participants (*p* = 0.038, 0.045, 0.018). There was no significant association between protein, vitamin, mineral and sleep quality (not shown in the table).

## Discussion

Our study reported that the vegetarians had better sleep quality (lower PSQI scores and higher sleep self-satisfaction ratio) than the omnivores. In both vegetarians and omnivores, the depression condition (CES-D score) was associated with sleep quality. After adjusting for depression condition, the difference in sleep quality between vegetarians and omnivores became insignificant. The findings reveal that being vegetarian is associated with better sleep quality, possibly due to lower levels of depression and other improved mental health. According to our results, mental health may be one of the mediating factors explaining the positive association of vegetarian diet with sleep quality in healthy adults. This provides evidence for the potential of vegetarian diets in the promotion of fine mental health and high-quality sleep.

The current study reported that after adjusting depression condition the difference in sleep quality between vegetarians and omnivores was not significant anymore. It indicates that the difference in sleep quality between these two populations might be moderated by depression condition. Our results are in line with other recent studies that have reported the effect of mental health on sleep^10,33^. In addition, people with mood disorders frequently experience difficulty falling and staying asleep and may represent circadian phenotypes (e.g., early morning awakening and day dysfunction)^34^. At a neurobiological level, the brain structures and neurochemicals involved in the regulation of emotion also manage sleep^35^, and nearly all affective disorders co-occur with sleep abnormalities^36^.

In the present study, we found that following a vegetarian diet was associated with less depression. This finding was in accordance with other studies regarding the association between being vegetarian and mental health. For instance, a cross-sectional study found that vegans experienced less stress and anxiety than omnivores^37^. A systematic review of 24 independent cohorts revealed that high intakes of fruits, vegetables, nuts and whole grains are inversely associated with depression^38^. All of these indicated that vegetarian dietary patterns may account for the effect of vegetarianism on improving mental health.

One of possible explanations for improving depression by vegetarian diets might be due to the anti-inflammatory effect of various antioxidants widely distributed in the plant food such as vegetables, fruits, grains, nuts, and soybeans^39,40^. In addition, not consuming meat might be another reason for the improved depression condition in vegetarians. In a large cohort study, Ford et al. found the positive association between meat consumption and depression condition, i.e. higher red meat intake was associated with higher depression score^41^. A meta-analysis consisting of 21 studies from ten countries also showed that low intakes of animal-based foods was associated with decreased risk of depression^42^. Higher fat content in animal food elevates the level of inflammatory cytokines, which lead to depression^43^. Similarly, a plan-based vegetarian diet reduces inflammatory markers, which could also explain the role of vegetarianism on sleep quality. Levels of pro-inflammatory markers (C-reactive protein and TNF-α) increase with poor quality sleep^44^.

Different factors were associated with the sleep quality in vegetarian or omnivore groups. In the vegetarian group, the influencing factors included sedentary time, skeletal muscle percentage, visceral fat area, and systolic blood pressure. In the omnivore group, the factors included age and working intensity. Since the contributing rate of these variable to sleep quality (unadjusted regressions) is much less than depression (Standardized β = 0.370), and these variable are not positive in all diet groups, we regard mental health as the most vital factor affecting sleep in this study. Furthermore, in accordance with previous studies^12,14^, we also found high-carbohydrate and high-fat intakes were independently associated with poor sleep quality. However, the significant association was only in all participants, but not in either vegetarian or omnivore group, which could be explained by the halved sample size, and the small contributions of these nutrients to sleep quality.

To the best of our knowledge, this was the first study to investigate the difference in sleep quality between vegetarian and omnivores among healthy population. This is a paired design study that participants in the control group was consist of mostly omnivorous relatives of the participants in the vegetarian group with similar characteristics and lifestyles, to minimize the influence of confounding factors. Moreover, important confounders of sleep have been assessed, including use of substances such as alcohol, caffeine, tobacco, nutrients intakes and physical activity patterns, which gives our research more credibility. There were also several limitations to the study. First, our participants were volunteers recruited by convenience sampling, which might not represent the general vegetarian adults. Second, this study was conducted in one city of China—the generalizability of results to other regions of the world is limited. Third, the study design was cross-sectional, and any cause-and-effect relationship between sleep quality and vegetarian diet or depression condition and vegetarian diet was undetermined. Because of that, we can’t verify from this study that the difference in sleep quality between vegetarians and omnivores is entirely caused by depression condition. Similarly, we can't verify that the better sleep quality is caused by the adoption of a vegetarian diet. Thus, randomized controlled trails and cohort studies are urgently required to confirm vegetarian as a strategy to benefit psychological health and sleep quality.

In conclusion, our findings revealed that the sleep quality and depression condition of the vegetarian population are better than those of the omnivores. However, a vegetarian diet might not have a direct effect on sleep quality, rather indirectly influence it through its beneficial effect on mental health in vegetarians.

## Data Availability

The datasets used and/or analyzed during the current study are available from the corresponding author on reasonable request.

## References

[1] Krueger JM, Frank MG, Wisor JP, Roy S (2016). Sleep function: Toward elucidating an enigma. Sleep Med. Rev..

[2] Kloss JD, Perlis ML, Zamzow JA, Culnan EJ, Gracia CR (2015). Sleep, sleep disturbance, and fertility in women. Sleep Med. Rev..

[3] Chattu VK (2018). The global problem of insufficient sleep and its serious public health implications. Healthc. Basel.

[4] Ikehara S (2009). Association of sleep duration with mortality from cardiovascular disease and other causes for Japanese men and women: The JACC study. Sleep.

[5] Huang Y, Zhao N (2020). Generalized anxiety disorder, depressive symptoms and sleep quality during COVID-19 outbreak in China: A web-based cross-sectional survey. Psychiat. Res..

[6] Troynikov O, Watson CG, Nawaz N (2018). Sleep environments and sleep physiology: A review. J. Therm. Biol..

[7] Van Cauter E, Leproult R, Plat L (2000). Age-related changes in slow wave sleep and REM sleep and relationship with growth hormone and cortisol levels in healthy men. JAMA.

[8] Kansagra S (2020). Sleep disorders in adolescents. Pediatrics.

[9] Yang Y, Shin JC, Li D, An R (2017). Sedentary behavior and sleep problems: A systematic review and meta-analysis. Int. J. Behav. Med..

[10] Ptáǒek LJ, Fu YH, Krystal AD (2016). Sleep and mood: Chicken or egg?. Biol. Psychiat..

[11] Hoffman SR, Stallings SF, Bessinger RC, Brooks GT (2013). Differences between health and ethical vegetarians. Strength of conviction, nutrition knowledge, dietary restriction, and duration of adherence. Appetite.

[12] Lindseth G, Murray A (2016). Dietary macronutrients and sleep. West J. Nurs. Res..

[13] Grandner MA, Kripke DF, Naidoo N, Langer RD (2010). Relationships among dietary nutrients and subjective sleep, objective sleep, and napping in women. Sleep Med..

[14] St-Onge MP, Mikic A, Pietrolungo CE (2016). Effects of diet on sleep quality. Adv. Nutr..

[15] Zhou J, Kim JE, Armstrong CL, Chen N, Campbell WW (2016). Higher-protein diets improve indexes of sleep in energy-restricted overweight and obese adults: Results from 2 randomized controlled trials. Am. J. Clin. Nutr..

[16] Binks H, Grace EV, Gupta C, Irwin C, Khalesi S (2020). Effects of diet on sleep: A narrative review. Nutrients.

[17] Craig WJ (2010). Nutrition concerns and health effects of vegetarian diets. Nutr. Clin. Pract..

[18] Daneshzad E (2020). Association of dietary acid load and plant-based diet index with sleep, stress, anxiety and depression in diabetic women. Br. J. Nutr..

[19] Tsai PS (2005). Psychometric evaluation of the Chinese version of the pittsburgh sleep quality index (CPSQI) in primary insomnia and control subjects. Qual. Life Res..

[20] Buysse DJ, Reynolds CF, Monk TH, Berman SR, Kupfer DJ (1989). The pittsburgh sleep quality index: A new instrument for psychiatric practice and research. Psychiat. Res..

[21] Radloff LS (1977). The CES-D scale: A self-report depression scale for research in the general population. Appl. Psychol. Meas..

[22] Sun XY, Li YX, Yu CQ, Li LM (2017). Reliability and validity of depression scales of Chinese version: A systematic review. Zhonghua Liu Xing Bing Xue Za Zhi.

[23] Yang L, Jia CX, Qin P (2015). Reliability and validity of the center for epidemiologic studies depression scale (CES-D) among suicide attempters and comparison residents in rural China. BMC Psychiat..

[24] Chen CM (2008). Overview of obesity in mainland China. Obes. Rev..

[25] Li LM (2005). A description on the Chinese national nutrition and health survey in 2002. Zhonghua Liu Xing Bing Xue Za Zhi.

[26] Institute of Nutrition and Health (2018). China Food Composition Tables.

[27] Wells VE, Deykin EY, Klerman GL (1985). Risk factors for depression in adolescence. Psychiat. Dev..

[28] Opie RS, O'Neil A, Itsiopoulos C, Jacka FN (2015). The impact of whole-of-diet interventions on depression and anxiety: A systematic review of randomised controlled trials. Public Health Nutr..

[29] Kim BI (2018). Factors related with quality on sleep of daytime workers. Ann. Occup. Environ. Med..

[30] Wang F, Bíró É (2021). Determinants of sleep quality in college students: A literature review. Explor. NY.

[31] Veronda AC, Irish LA, Delahanty DL (2020). Effect of smoke exposure on young adults' sleep quality. Nurs. Health Sci..

[32] Ohayon MM, Carskadon MA, Guilleminault C, Vitiello MV (2004). Meta-analysis of quantitative sleep parameters from childhood to old age in healthy individuals: Developing normative sleep values across the human lifespan. Sleep.

[33] Palmer CA, Alfano CA (2017). Sleep and emotion regulation: An organizing, integrative review. Sleep Med. Rev..

[34] Schotte CK, Maes M, Cluydts R, Cosyns P (1997). Cluster analytic validation of the DSM melancholic depression. The threshold model: integration of quantitative and qualitative distinctions between unipolar depressive subtypes. Psychiat. Res..

[35] Goldstein AN, Walker MP (2014). The role of sleep in emotional brain function. Annu. Rev. Clin. Psychol..

[36] Harvey AG (2011). Sleep and circadian functioning: Critical mechanisms in the mood disorders?. Annu. Rev. Clin. Psychol..

[37] Beezhold B, Radnitz C, Rinne A, DiMatteo J (2015). Vegans report less stress and anxiety than omnivores. Nutr. Neurosci..

[38] Molendijk M, Molero P, Ortuño Sánchez-Pedreño F, Van der Does W, Angel Martínez-González M (2018). Diet quality and depression risk: A systematic review and dose-response meta-analysis of prospective studies. J. Affect. Disord..

[39] McMartin SE, Jacka FN, Colman I (2013). The association between fruit and vegetable consumption and mental health disorders: Evidence from five waves of a national survey of Canadians. Prev. Med..

[40] Slavich GM, Irwin MR (2014). From stress to inflammation and major depressive disorder: A social signal transduction theory of depression. Psychol. Bull..

[41] Ford PA, Jaceldo-Siegl K, Lee JW, Youngberg W, Tonstad S (2013). Intake of Mediterranean foods associated with positive affect and low negative affect. J. Psychosom. Res..

[42] Li Y (2017). Dietary patterns and depression risk: A meta-analysis. Psychiat. Res..

[43] Dutheil S, Ota KT, Wohleb ES, Rasmussen K, Duman RS (2016). High-fat diet induced anxiety and anhedonia: Impact on brain homeostasis and inflammation. Neuropsychopharmacology.

[44] Cha E, Talman MS, Massey AH, Yan F, Rogers AE (2018). Sleep, lifestyle behaviors, and cardiometabolic health markers in overweight/obese young adults: A pilot study using the sensewear^®^ Armband. Biol. Res. Nurs..

